# Porous-Cladding Polydimethylsiloxane Optical Waveguide for Biomedical Pressure Sensing Applications

**DOI:** 10.3390/s25144311

**Published:** 2025-07-10

**Authors:** Koffi Novignon Amouzou, Alberto Alonso Romero, Dipankar Sengupta, Camila Aparecida Zimmermann, Aashutosh Kumar, Normand Gravel, Jean-Marc Lina, Xavier Daxhelet, Bora Ung

**Affiliations:** Department of Electrical Engineering, École de Technologie Supérieure, 1100 Notre-Dame Street West, Montreal, QC H3C 1K3, Canada; koffi-novignon.amouzou.1@ens.etsmtl.ca (K.N.A.); alberto.alonso-romero.1@ens.etsmtl.ca (A.A.R.); dipankar.sengupta@etsmtl.ca (D.S.); camila-aparecida.zimmermann@ens.etsmtl.ca (C.A.Z.); aashutosh.kumar@etsmtl.ca (A.K.); normand.gravel@etsmtl.ca (N.G.); jean-marc.lina@etsmtl.ca (J.-M.L.); xavier.daxhelet@etsmtl.ca (X.D.)

**Keywords:** Polydimethylsiloxane, waveguide, pressure sensor, microbubbles, total internal reflection, frustrated total internal reflection

## Abstract

We report a new concept of a pressure sensor fully made from polydimethylsiloxane with a solid core and porous cladding that operates through (frustrated) total internal reflection. A flexible and sensitive rectangular cross-section waveguide was fabricated via the casting and molding method. The waveguide’s optical losses can be temperature-controlled during the fabrication process by controlling the quantity of microbubbles incorporated (2% approximately for samples made at 70 °C). By controlling the precuring temperature, the microbubbles are incorporated into the waveguides during the simple and cost-effective fabrication process through the casting and molding method. For these samples, we measured good optical loss tradeoff of the order of 1.85 dB/cm, which means that it is possible to fabricate a solid-core/clad waveguide with porous cladding able to guide light properly. We demonstrated the microbubble concentration control in the waveguide, and we measured an average diameter of 239 ± 16 µm. A sensitivity to pressure of 0.1035 dB/kPa optical power loss was measured. The results show that in a biomedical dynamic pressure range (0 to 13.3 kPa), this new device indicates the critical pressure threshold level, which constitutes a crucial asset for potential applications such as pressure injury prevention.

## 1. Introduction

Nowadays, the development of pressure and shear sensor platforms arouses strong interest for a variety of applications, notably for the biomedical industry [1,2,3,4,5,6,7,8,9,10,11]. Existing pressure sensor technologies on the market can be classified in two general categories, namely electronic and optical technology. In general, pressure-mapping technologies based on flexible electronic systems (e.g., piezoresistive [12,13,14,15,16], capacitive [17,18,19,20,21,22], piezoelectric, triboelectric [23,24,25,26,27,28], electrochemical pressure sensors [29,30,31,32]) are reliable and accurate; however, they can be expensive (especially for 2D mapping applications) and may require frequent calibration. Moreover, all types of flexible pressure sensors are prone to damage over time and show limitations in the operating range and their sensitivity [33,34]. Optical-based pressure devices have the potential to enhance service life and enable the optical multiplexing capabilities of several sensors while being less susceptible to electromagnetic interferences [5,34]. There is an increasing need for a variety of compact, low-power, mechanically robust, flexible, and wearable sensors that can monitor pressure and shear stress. For biomedical, athletic, and consumer applications, the development of flexible optical pressure sensors requires the use of specialized materials such as elastomeric materials with good optical properties. Recently, much progress has been made in the development of biomedical optical waveguide sensors [8,35,36,37].

Elastomeric materials are promising for the manufacturing of innovative pressure and shear sensors due to their acceptable thermo-mechanical and optical properties [38,39,40]. These materials offer the advantage of manufacturing optical waveguides whose dimensions are of the order of a millimeter, which constitutes an asset for better coupling with LED sources. They are also very resistant to water, easily conformable to complex shapes (skin, teeth, bones, joints, etc.), and have good biocompatibility and thermo-mechanical stability. Moreover, optical elastomers are amenable to the introduction of photosensitive dopants, pigments and dyes [41,42,43,44], or microstructures [45,46,47] into their polymer matrix, and they are easy to process, mold, and handle. In particular, polydimethylsiloxane (PDMS), which is part of the family of silicones, is a promising material for the development of flexible waveguide sensors that operate in the visible and near-infrared spectral range [48]. PDMS waveguides can be highly responsive towards physical deformations (external pressure, bending, stretching) measured through the intensity variation in photons detected at the end of these.

In this paper, we present a new type of optical waveguide-based pressure sensor consisting of a rectangular cross-section PDMS waveguide that incorporates some porosity in its structure. The porosity of the waveguide is created by the precise incorporation of air microbubbles during the fabrication process. Our study demonstrates a new concept of a flexible and stretchable optical waveguide obeying the principles of guiding light by the classical total internal reflection and frustrated total internal reflection. The transverse compression tests indicate that the proposed sensing platform is sensitive enough for biomedical applications that monitor pressure values at and above the blood capillary pressure level (i.e., 4.3 kPa (32 mmHg)), which is key to prevent local tissue hypoxia, which eventually leads to debilitating pressure injuries [49,50,51].

## 2. Sensor Structure and Optical Sensing Mechanism

**Figure 1:**
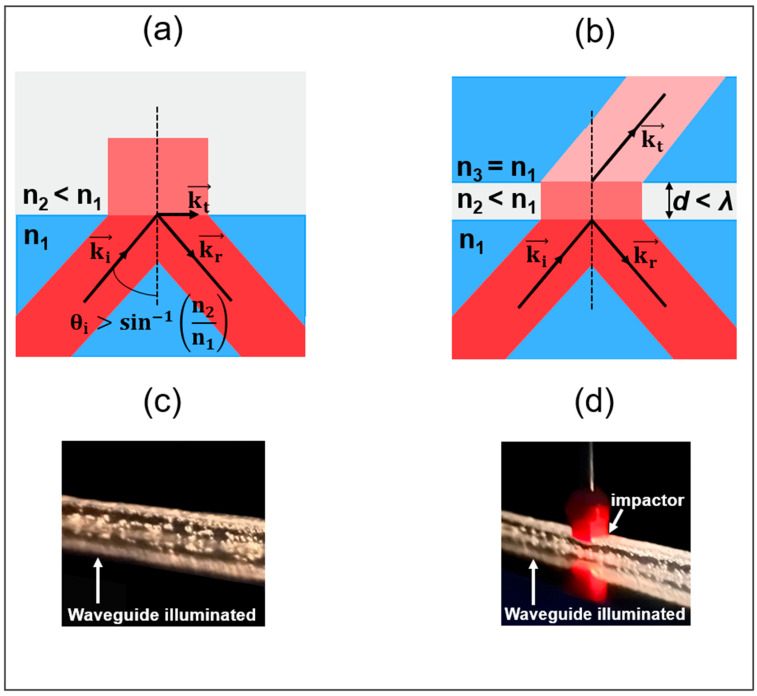
Illustrations of (**a**) the total internal reflection (TIR) between a prism and air and (**b**) the frustrated total internal reflection (FTIR) by bringing a second prism in the near-field of the first one ($n_{1}$, $n_{2}$, and $n_{3}$ are the refractive indices of the media; $\vec{k_{i}}$, $\vec{k_{r}}$, $\vec{k_{t}}$ represent the incident, reflected, and transmitted wave vectors, respectively; and $\theta_{i}$ is the incidence angle) [52]. Side views of a porous-clad waveguide illuminated by a light source at the proximal end (**c**) without external compression and (**d**) under strong external compression (8 kPa).

**Figure 2:**
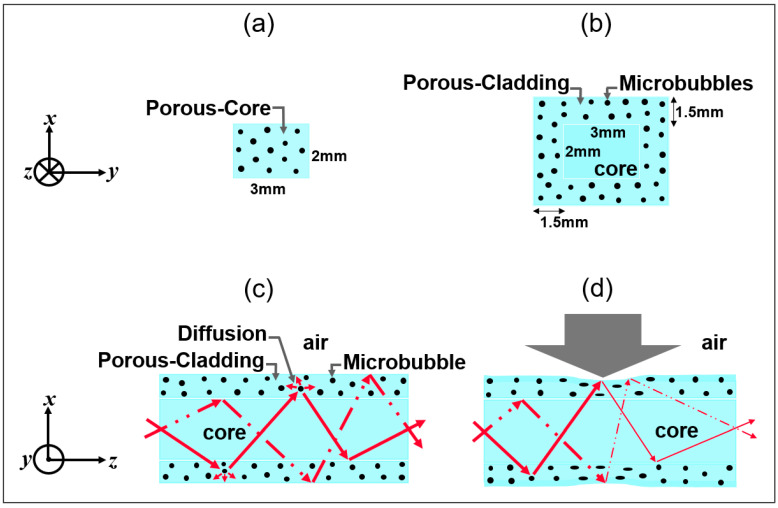
(**a**,**b**) Porous waveguide transverse cross-section; longitudinal cross-section of core/porous-cladding waveguide: (**c**) undeformed and (**d**) under compression. Arrows represent propagation of light rays.

Light guiding in PDMS optical waveguides usually occurs through the phenomenon of total internal reflection (TIR), as shown in Figure 1a. When the refractive index of the core medium becomes very close to that of the cladding, the reflectivity of the interface is lowered so that wave guiding occurs via frustrated total internal reflection (FTIR) [52,53], as illustrated in Figure 1b. The sensor studied in this paper is a flexible waveguide completely made with a single type of PDMS material. Air bubble microstructures are introduced into the polymer matrix during the manufacturing process. To characterize this new optical structure, a porous-core/air-clad waveguide (Figure 2a) was fabricated. This allowed us to better understand how to control the incorporation of microbubbles in PDMS material and their effect on light guiding properties. A rectangular (2 mm × 3 mm) core cross-section design was chosen for its manufacturing simplicity and also to allow us to study the effect of the transverse mechanical deformation for different material thicknesses, as similarly performed in a prior study [48]. The second design (Figure 2b) consists of a solid PDMS core (2 mm × 3 mm) surrounded by a 1.5 mm thick porous cladding containing a specific concentration of air microbubbles. The cladding thickness was chosen in order to approach the lower manufacturing limit of the in-house casting and molding setup, which uses CNC machined aluminum molds. Microbubbles are added as light-diffusing points in the waveguide’s cladding. When the waveguide is compressed, these diffusion points are eliminated such that there is a TIR that occurs at the outer cladding and air boundary, while FTIR occurs between the solid core and the porous-cladding boundary (Figure 2d), owing to the imperfection related to the fabrication process, which is described in the next section. While the exact proportions of TIR and FTIR are not known, our results indicate that FTIR waveguiding dominates for the proposed porous-clad waveguide design. Indeed, we observed that the injected light remains well confined within the waveguide’s core region (see side view in Figure 1c and output waveguide core imaging shown further in Section 4.2), even though both the core and cladding are fabricated with a single material of the same refractive index (not counting the much larger microbubbles compared to the wavelength). Furthermore, when the waveguide is slightly compressed (corresponding to ZONES 1 and 2 defined in Section 4.2), the suppression of the diffusing microbubbles enables us to enhance the waveguide’s transmission by allowing part of the transmitted waves in the FTIR process (represented by $\vec{k_{t}}$ in Figure 1b) to undergo TIR at the outer cladding–air boundary and to return in the undeformed core. The latter phenomenon is supported by experimental results presented in Section 4.2, where we observe an enhancement of the optical transmission at small compression values (in ZONES 1 and 2) before undergoing a decrease in transmission (in ZONE 3) that is attributed to the onset of the core’s deformation and radiative losses. For sufficiently strong core deformations, one can observe the radiated light escaping from the side of the waveguide (Figure 1d).

## 3. Materials and Methods

### 3.1. Waveguide Fabrications: Porous-Core/Air-Clad and Solid-Core/Porous Cladding

A solvent-free method for manufacturing PDMS porous waveguides was developed. First, a rectangular core cross-section (2 × 3 mm^2^ cross-section) waveguide 20 cm long was fabricated using PDMS Sylgard 184 at a mixing ratio of base-to-curing agent of 20:1 (Dow Corning, Midland, MI, USA). The 20:1 mixing ratio was used in this study to ensure adequate mechanical flexibility of the waveguide and good sensitivity of the latter to compression. Prior works indicate that similar PDMS waveguides fabricated using the same mixing ratio (i.e., 20:1) are robust and can support up to 75% transverse deformation before irreversible structural damage [48,54,55].

**Figure 3:**
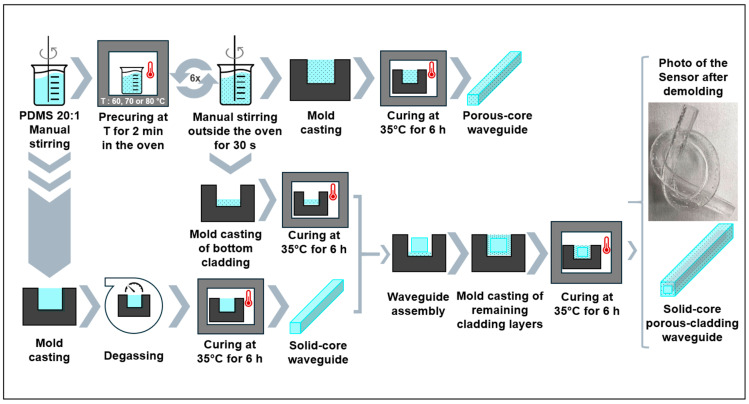
Porous waveguide manufacturing steps (70 °C precuring) showing the photo of the waveguide after demolding and 24 h of relaxation.

To fabricate the porous-core/air-clad waveguide, the PDMS mixture was put in the oven for precuring for two minutes before being stirred again for thirty seconds; finally, it was put back in the oven for two more minutes. This process was repeated without prior degassing until fifteen minutes of precuring time was reached. The resulting mixture was cast in a metal mold and left to cure at 35 °C for six hours, after which the porous-core/air-clad waveguide was demolded (Figure 3). The size of the porous-core/air-clad waveguide was 2 × 3 mm^2^ in transverse cross-section and 20 cm in length.

To obtain the solid-core/porous-cladding waveguide, we first fabricated the solid core using PDMS Sylgard 184 (Dow Corning, Midland, MI, USA) at a mixing ratio of base-to-curing agent of 20:1 through the casting and molding method (including a degassing step to remove all air bubbles); we then covered it on all sides with a 1.5 mm layer of porous PDMS using the multi-step procedure illustrated in Figure 3. After the polymer mix (PDMS base to curing agent of 20:1 mix ratio) was stirred and precured at 70 °C during a cycle lasting fifteen minutes, a first layer of porous PDMS was cast at the bottom of the mold. Then, the solid core was placed on top of the bottom porous layer, and the sides of the waveguide were subsequently filled with porous PDMS to create the remaining porous layers (Figure 3). The system was left to cure at 35 °C for six hours, after which the solid-core/porous-cladding waveguide was demolded. The final size of the solid-core/porous-cladding waveguide was 5 × 6 mm^2^ in transverse cross-section and 20 cm in length.

### 3.2. Quantitative Analysis of Microbubbles

#### 3.2.1. Microbubble Effects on Waveguiding Optical Losses

**Figure 4:**
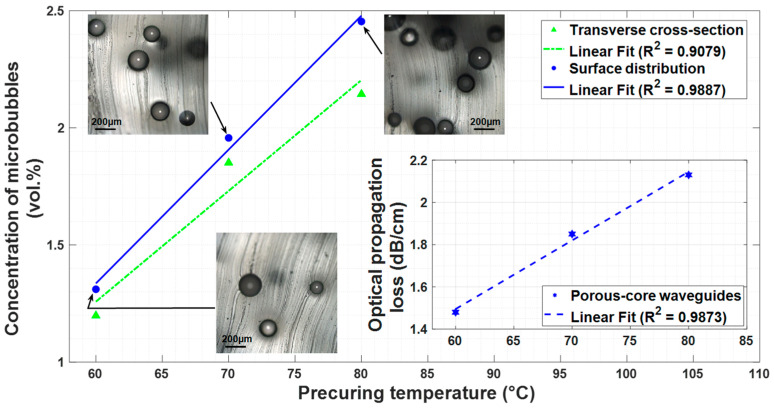
Optical propagation loss and microbubble quantity in a porous-core waveguide as a function of precuring temperature.

The simple manufacturing procedure allows us to control the volume concentration of air microbubbles in the PDMS matrix. The degree of control depends on the precuring temperature. Three different precuring temperatures (60 °C, 70 °C, and 80 °C) were tested in this study. The concentration of microbubbles present in the PDMS samples increases linearly (from 1.30 to 2.47% vol.) with the precuring temperature (from 60 to 80 °C), as shown in Figure 4. This result is expected considering the PDMS curing kinetics, whose gel point (i.e., the time at which the sol-to-gel transition occurs) decreases as the temperature increases, leading to a more efficient air bubble entrapment [56]. Surface images of the waveguides obtained using a microscope (LEXT OLS4000, Olympus, Japan) and shown in Figure 4 indicate that the air microbubbles are of spherical shape. The analysis of the quantity of microbubbles present on the sample surface and transverse cross-section shows an almost linear trend as a function of the precuring temperature whose $R^{2}$ values are, respectively, 0.9887 and 0.9079 (Figure 4). The microbubble’s concentration along the waveguide’s surface is slightly greater than that in the transverse cross-section (+9%, +6%, and +14%, respectively, at 60 °C, 70 °C, and 80 °C). Indeed, closer to the waveguide’s surface, there is a higher concentration of microbubbles because they tend to naturally migrate to the surface during the curing process where they are trapped. Optical propagation losses in the porous-core waveguides were measured by the cutback method (inset of Figure 4) and follow a similar linear ($R^{2}$ value of 0.9873) dependence with the precuring temperature and, therefore, the microbubble concentration. For the porous PDMS samples made at 70 °C (slightly less than 2% vol. of microbubbles), an optical propagation loss of 1.85 dB/cm was measured and found to be a suitable trade-off for subsequent tests that required small microporosity to enable sensing functionalities while remaining sufficiently transparent.

#### 3.2.2. Average Microbubble Diameter Measurements

**Figure 5:**
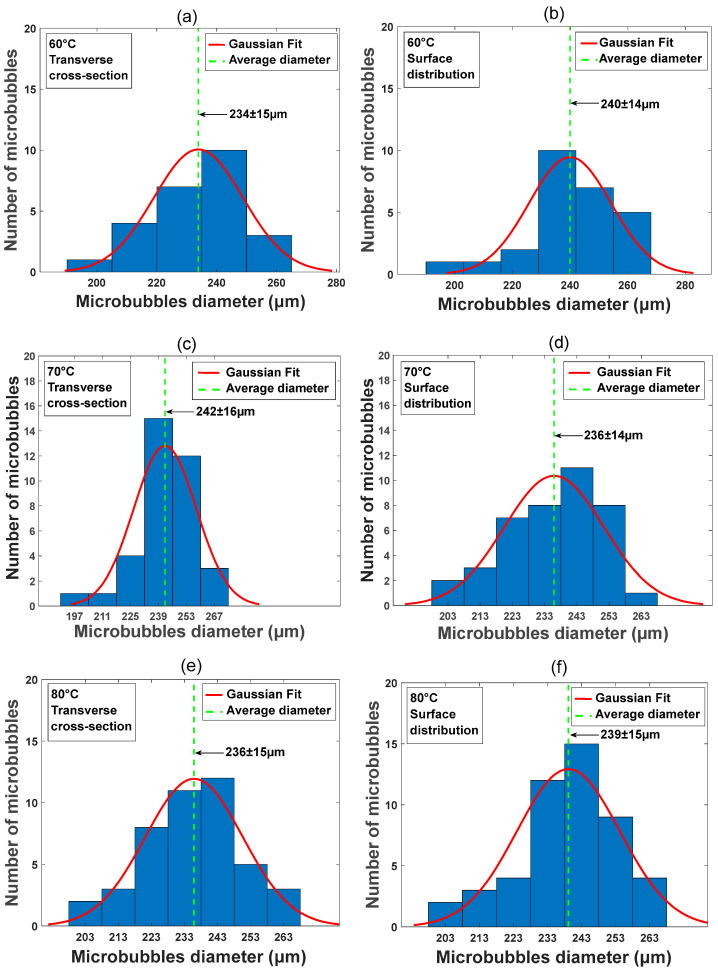
Porous-core waveguides: Statistical distribution of the microbubble number as a function of their average diameter at 60 °C (**a**,**b**), 70 °C (**c**,**d**), and 80 °C (**e**,**f**) precuring temperatures.

For each waveguide, transverse cross-section and surface distribution images (dimensions of 1280 µm × 1280 µm) were collected using the microscope (LEXT OLS4000, Olympus, Japan). Subsequently, the quantity and number of microbubbles along the waveguide surface and in the transverse cross-section for samples made at three different precuring temperatures (60 °C, 70 °C, and 80 °C) were evaluated. The latter was accomplished through a statistical distribution study (Figure 5) on the collected images by using Matlab’s image processing toolbox in order to find the number of circular microbubbles contained in the image considered and their corresponding diameters. The average diameter of the microbubbles was thus calculated via a Gaussian fit of the statistical distribution (Figure 5). For the samples made at 60 °C, 70 °C, and 80 °C, we measured an average microbubble diameter of 237 ± 15 µm (Figure 5a,b), 239 ± 16 µm (Figure 5c,d), and 238 ± 15 µm (Figure 5e,f), respectively. We therefore demonstrated that it is possible to control the concentration of microbubbles in the PDMS samples while keeping a relatively constant size distribution and average diameter. Using the same methodology, it is also possible to design and fabricate a solid-core waveguide cladded with porous PDMS, as described in the next section.

## 4. Characterization of Solid-Core/Porous-Cladding Waveguide

For the solid-core/porous-cladding waveguide fabrication, the 70 °C precuring temperature was used, which yielded approximately 2% of microbubbles by volume of the porous cladding. This level of microbubbles in the porous cladding was deemed acceptable to allow light to be partially transmitted in the porous cladding and enable the FTIR wave guiding. The size of the sensor is a 5 × 6 mm^2^ transverse cross-section and is 20 cm in length. An optical propagation loss of 1.28 dB/cm was measured for the solid-core/porous-cladding waveguide via the cutback method. This value of attenuation is larger than a comparable solid-core PDMS waveguide (0.37 dB/cm) [48] but can still enable optical sensing functionalities (as shown in Section 4.2). After 24 h of relaxation, the mechanical and optical characterizations were performed on the fabricated PDMS waveguides, as described in the next section.

### 4.1. Transverse Compression Experimental Setup

**Figure 6:**
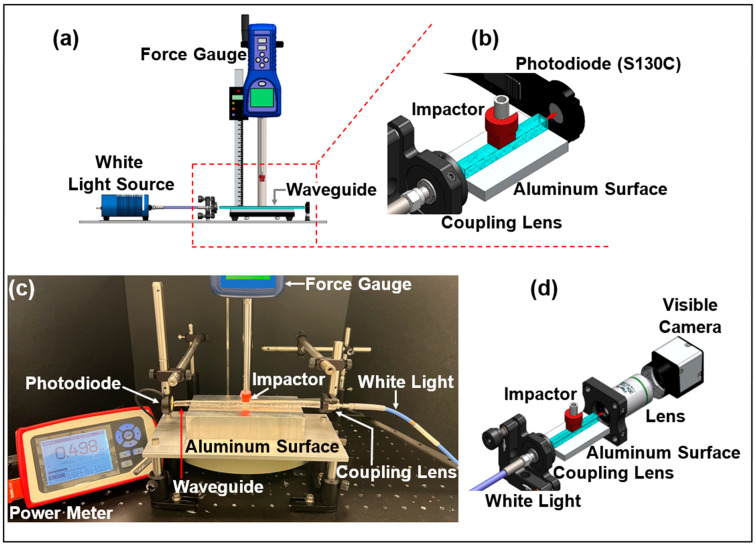
(**a**) Experimental setup used for transverse compression, (**b**) zoom image of the setup showing the coupling lens and the photodiode placed, respectively, at the beginning and end of the waveguide, (**c**) a photo of the experimental setup, and (**d**) the setup used for recording the waveguide’s core transmitted light images.

We performed a transverse compression test to measure the waveguide response to transverse deformation. For this test, a non-polarized white light (Ocean Optics HL-2000-HP Tungsten Halogen Light Source) was coupled to the solid core with a collimating lens (F230SMA-A, Thorlabs Inc., Newton, NJ, USA) at the proximal end, and a silicon photodiode (S130C, Thorlabs Inc., Newton, NJ, USA) was used to measure the total optical power at the waveguide output (Figure 6). The pressure sensing measurement modality is based on the analysis of the transmitted optical power in the waveguide. The waveguide was placed on a flat aluminum surface and deformed by applying forces ranging from 0 to 1 N on the top center of its cross-section with a rectangular impactor (7 mm × 15 mm) while monitoring the transmitted optical power (Figure 6a,b). Using a similar setup (Figure 6c), imaging of the transmitted light from the waveguide’s core was performed by means of a collimation lens at the waveguide output and a visible camera (EO-0413M, Monochrome Camera, Edmund Optics Inc., Barrington, NJ, USA). An inline optical attenuator was also used to prevent camera image saturation.

### 4.2. Results and Discussion

**Figure 7:**
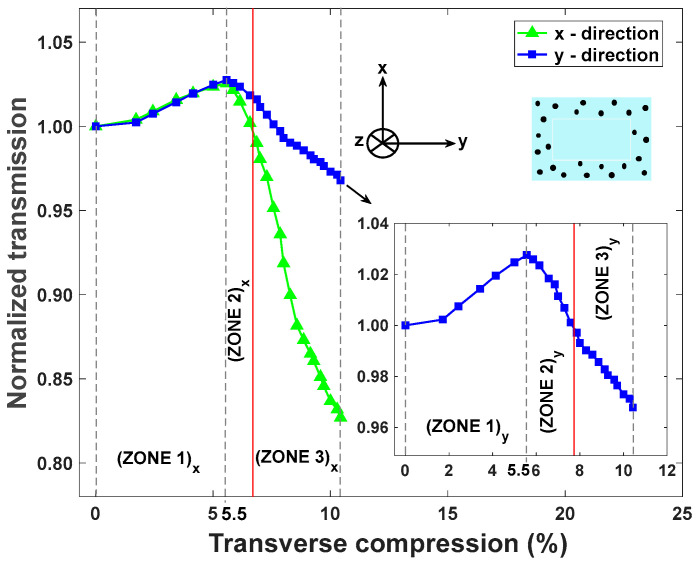
Normalized transmission measurement along the *x* and *y* (in inset) directions as a function of transverse compression (%). Red line indicates the boundary between ZONES 2 and 3.

The compression test results indicate that larger optical losses occur for compression along the thinner *x* direction compared to the *y* direction of the waveguide cross-section (Figure 7). Furthermore, the deformation of the porous cladding (ZONE 1: 0% to 5.5% compression) induces the suppression of the scattering microbubbles, resulting in an increase in the transmitted optical power, while the transition from FTIR waveguiding to TIR waveguiding occurs (Figure 7). The white light intensity increases by approximately 3% to a maximum value above the initial value for a compression level of around 5.5%. When the compression reaches 5.5%, we note the transition between the porous-cladding deformation and the beginning deformation of the shape of the core (ZONE 2: 5.5% to 6.5% compression), which is accompanied by a decrease in the transmitted optical power up to the normalized initial value (Figure 7). From 6.5% compression and onwards (ZONE 3), the solid core gradually deforms, which is accompanied by a decrease in the transmitted optical power. Due to the different thicknesses at *x* and *y* directions of the waveguide’s core, we have definitions of ZONES 1, 2, and 3, which are shifted. These results indicate that the device can operate as a pressure sensor in ZONE 3. However, there is ambiguity in ZONES 1 and 2 because the measured transmission value corresponds to two distinct values of applied pressures, which prevents the device from acting as a quantitative pressure sensor for the range of transverse compressions located in ZONES 1 and 2.

**Figure 8:**
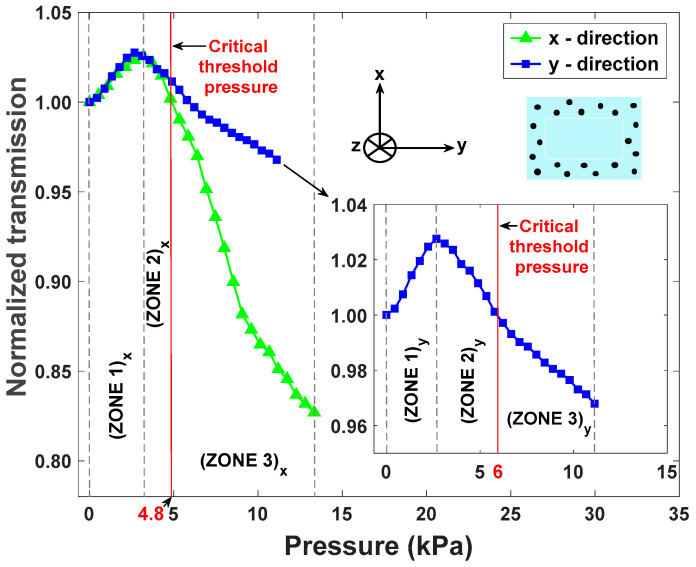
Normalized transmission measurement along the *x* and *y* (in inset) directions as a function of pressure applied (kPa). Red line indicates the boundary between ZONES 2 and 3.

During sample deformation in the *x* and *y* directions, the top surfaces covered by the impactor are 90 mm^2^ and 75 mm^2^ respectively. Pressure values in kPa are thus defined by considering the applied force in N units over the overlap area of the impactor’s surface with the waveguide’s cross-section. All the tests were performed in a pressure sensing dynamic range of 0 to 13.3 kPa (Figure 8) by considering the applied force and impacted surface, which indicate that such a sensor can monitor the compression condition of individuals. In this study, the critical pressure level required to monitor the compression status of patients at risk to develop pressure injuries is taken as the blood capillary pressure, which is of the order of 4.3 kPa (32 mmHg) [49]. When the sensor signal is in ZONES 1 and 2, the compression level remains in the qualitative “safe zone”. However, when the optical transmission signal is in ZONE 3, it indicates that the exerted pressure is above the critical threshold level (>32 mmHg), where a person is at risk of developing pressure injuries if no pressure relief is provided in a timely manner. Furthermore, when the signal is in ZONE 3, the device operates in a quantitative manner by providing direct measurement of the applied pressure as a function of optical transmission (see Figure 8). Along *x* and *y* directions, the maximum transmission values correspond to pressure values of 3.2 kPa and 2.7 kPa, respectively. The critical threshold pressure levels in both *x* and *y* directions are, respectively, 4.8 kPa and 6.0 kPa, which are very close to the typical value of capillary blood pressure estimated at 4.3 kPa (32 mmHg) [49], which confirms the potential of this sensor to be used for pressure monitoring in biomedical applications.

**Figure 9:**
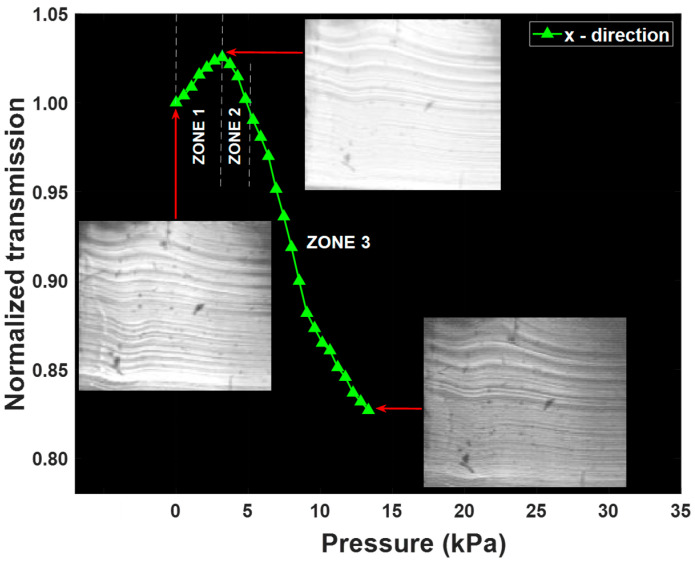
Output light imaging of the waveguide core versus the normalized transmission measurement along *x* directions as a function of pressure applied (kPa).

As expected, with 3.2 kPa pressure applied along the *x* direction, we note and observe an increase in output intensity from the waveguide core, as shown in Figure 9. For a high-pressure value tested at 13.3 kPa, we similarly observe a decrease in the output intensity, as indicated in Figure 2d, which confirms the previous result measured with the photodiode. We observe similar results along the *y* direction.

**Figure 10:**
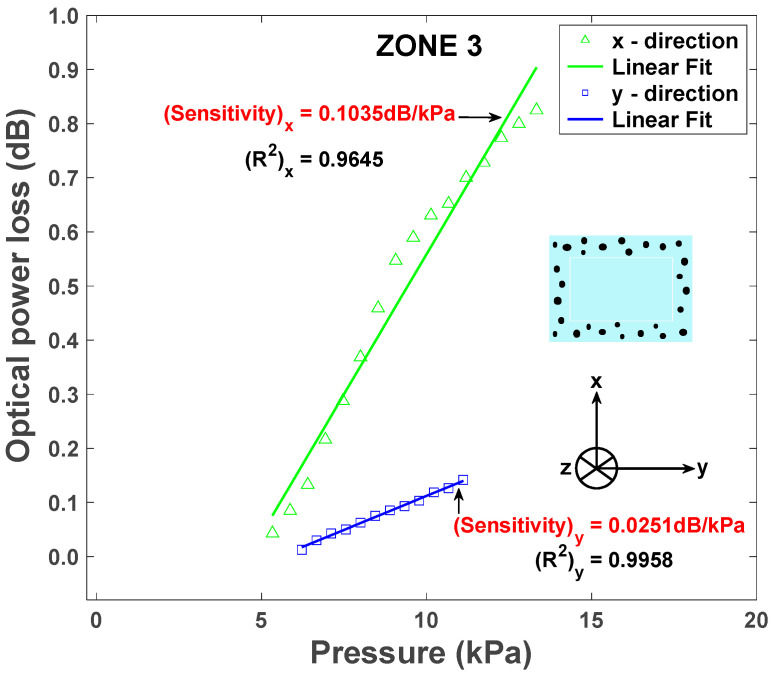
Sensor sensitivity measurement along the *x* and *y* directions defined as the slope of the relationship between the optical power loss and the pressure applied.

For optical transmission values inside ZONES 1 and 2, the device operates in qualitative mode and indicates that applied pressures are in a relatively “safe zone”. For transmission values in ZONE 3, the device operates in quantitative mode and can provide a measurement of the pressure applied. For the “quantitative mode of operation” in ZONE 3, the sensitivity of the sensor represents the slope of the linear fit of optical power loss versus pressure applied. We measured the $R^{2}$ fit of 0.9645 and 0.9958 in the *x* and *y* directions, respectively. Pressure sensitivities of 0.1035 dB/kPa and 0.0251 dB/kPa optical losses were measured along the *x* and *y* directions, respectively (Figure 10). In units of kPa^−1^, the sensitivity of the proposed device is 0.1074 kPa^−1^ in the range of 5.3 to 13.3 kPa, which is comparable with a sensitivity value of 0.1570 kPa^−1^ in the range of 0 to 6.0 kPa reported in [57].

As explained above, a difference in sensitivity values is related to the thickness of the core along the *x* and *y* directions. We note that the rectangular shape of the waveguide’s cross-section was specifically selected in this study to investigate the role of the cross-section’s thickness on the sensitivity of the device. The results (Figure 8) indicate that thinner optical waveguides (i.e., pressure along the *x*-axis in this work) result in more sensitive pressure sensors. One can thus expect that a symmetric design (e.g., square or circular cross-section) will remove this anisotropic response and promote a uniform sensor response for any orientation of the plane of pressure application, which could be beneficial for real-world applications.

The response time of the sensor is defined as the time to reach 90% of the transmitted optical power by using the finger pressing–raising method [11]. In the *x* direction, the measured response time is 97 ms, while in the *y* direction, it is 114 ms. The recovery time of the device is defined as the time required to return to 90% of the initial transmitted optical power value (i.e., transmitted optical power value without any deformation). The corresponding measured recovery times are 130 ms and 148 ms, respectively, in the *x* and *y* directions. The measured response and recovery times of the proposed device are thus in the same range as with prior reported values for comparable porous structured pressure sensors that showed response times of 160 ms and 119 ms, as shown in [58,59], and a recovery time of 59 ms in [59].

A series of 12 repetitive transverse compression tests were performed to evaluate the repeatability of the sensitivity sensor in ZONE 3 along the *x* direction. The compression tests yielded an average sensitivity of 0.1105 ± 0.0218 dB/kPa. This latter result translates into ±2% deviation in sensitivity in ZONE 3 along the *x* direction, which indicates a relatively good repeatability in device sensitivity. Similar results are expected along the *y* direction.

The results are also reproducible for another waveguide prepared in the same manner. We note that the physical integrity of the waveguide was maintained even after hundreds of tests were performed, which reflects the robustness of the device. A previous study [48] indicates that similar PDMS waveguides can support up to 75% (53.3 kPa) transverse compression before irreversible structural damage. We expect the waveguide to be less resistant to elongation compared to a fully PDMS solid waveguide (stretchable up to 160%, as reported recently [48]), owing to the presence of the trapped bubbles that would make the sample more fragile to rupture.

## 5. Conclusions

We propose and demonstrate for the first time the fabrication of a sensitive optical pressure sensor completely made from PDMS with a solid core and a porous cladding that guides light via a total internal reflection and frustrated total internal reflection waveguiding mechanism. We show that the optical losses induced by the incorporation of microbubbles inside the cladding can be precisely tuned via precuring temperature control. The ensuing porous-cladding waveguide shows a sensitivity to pressure on the order of 0.1035 dB/kPa optical loss measured in a dynamic range of 0 to 13.3 kPa pressure values, which is acceptable for biomedical use. We also measured an optical propagation loss of 1.28 dB/cm and demonstrated the possibility to use this pressure sensor for critical pressure threshold level monitoring in biomedical applications towards the prevention of pressure injuries. This proposed device is relevant for pressure sensing, which is promising for biomedical optical sensing wearable sensor applications. In the future, it would be interesting to study the effect of waveguide dimensions on sensor sensitivity using waveguides with different core thicknesses. Potentially, the thickness of the device could be tuned to control the sensitivity of the waveguide sensor, while the porosity of the cladding can be tuned to determine the critical threshold pressure value. Also, further investigation of the role of the main fabrication parameters (i.e., curing temperature and the mixing ratio of base-to-curing agent) would enhance control over the size and shape of microbubbles, as well as their spatial distribution in the waveguide. Other methods of producing microbubbles in the waveguide and potentially controlling the geometry and size of the microbubbles during the process could also be explored. One alternative is to pre-manufacture the microbubbles using 3D printing technology [60] and then incorporate them into the cladding during the fabrication process. In this case, the choice of a printable polymer that is compatible with PDMS in terms of optical and chemo-mechanical bonding must be researched.

## Data Availability

Data are contained within the article.
